# Evaporation Estimation of Rift Valley Lakes: Comparison of Models

**DOI:** 10.3390/s91209603

**Published:** 2009-12-01

**Authors:** Assefa M. Melesse, Wossenu Abtew, Tibebe Dessalegne

**Affiliations:** 1 Department of Earth and Environment, Florida International University, 11200 SW 8th Street, Miami, FL 33199, USA; 2 South Florida Water Management District, 3301 Gun Club Road, West Palm Beach, FL 33406, USA; E-Mail: wabtew@sfwmd.gov; 3 BEM Systems Inc., 500 Australian Avenue South, Suite 616, West Palm Beach, FL 33401, USA; E-Mail: tdessalegne@bemsys.com

**Keywords:** evaporation, evapotranspiration, simple method, remote sensing, surface energy balance, Rift Valley Lakes

## Abstract

Evapotranspiration (ET) accounts for a substantial amount of the water flux in the arid and semi-arid regions of the World. Accurate estimation of ET has been a challenge for hydrologists, mainly because of the spatiotemporal variability of the environmental and physical parameters governing the latent heat flux. In addition, most available ET models depend on intensive meteorological information for ET estimation. Such data are not available at the desired spatial and temporal scales in less developed and remote parts of the world. This limitation has necessitated the development of simple models that are less data intensive and provide ET estimates with acceptable level of accuracy. Remote sensing approach can also be applied to large areas where meteorological data are not available and field scale data collection is costly, time consuming and difficult. In areas like the Rift Valley regions of Ethiopia, the applicability of the Simple Method (Abtew Method) of lake evaporation estimation and surface energy balance approach using remote sensing was studied. The Simple Method and a remote sensing-based lake evaporation estimates were compared to the Penman, Energy balance, Pan, Radiation and Complementary Relationship Lake Evaporation (CRLE) methods applied in the region. Results indicate a good correspondence of the models outputs to that of the above methods. Comparison of the 1986 and 2000 monthly lake ET from the Landsat images to the Simple and Penman Methods show that the remote sensing and surface energy balance approach is promising for large scale applications to understand the spatial variation of the latent heat flux.

## 1. Introduction

In most cases, evapotranspiration (ET) is the greatest consumer of the water budget, accounting for an average of 70 percent of the consumption of annual precipitation in the United States, and up to 95 percent in arid climates [1]. The magnitude of ET impacts the available water in a hydrologic system affecting water yield, storage and stream flows. Evapotranspiration is also one of the hydrologic parameters that has least variation on annual basis. Variation of ET at smaller timescales can be also very high. It is a major component of the water cycle and important in water resource development and management. Measurement and estimation of this parameter usually provides different results. Evapotranspiration estimation models range from the complex as Penman-Monteith method to the simple Pan method. Complex models require many meteorological measured and estimated input parameters which incur high monitoring cost. The error in measurement and estimation of input parameters increase the error in ET estimation. The adaptability of simpler methods, especially in geographical areas where there is limited resource for monitoring is worth investigation.

Traditional means for point ET estimation include the pan, Bowen ratio, eddy correlation, and aerodynamic techniques. It has been found that these methods are costly, time consuming, and require elaborate and sensitive measurement equipment [2]. For land surfaces, a root zone soil water balance approach based on water budget is also a technique used to estimate ET as a residual variable. Quantifying each component of the soil water balance is less appealing in terms of time, labor and money requirements. The lysimeter instrumentations are relatively simpler but are usually limited to research applications. While these traditional methods estimate ET at a point basis, recent methods have found success using remotely-sensed imagery for estimates at various spatial scales [3-15].

Unlike the above point measurements, remote sensing has the capacity to instantaneously acquire spectral signatures for large areas of the watershed and infer land-cover, vegetation cover, emissivity, albedo, surface temperature and energy flux information. Remote sensing approach has also proven to have regional applications and allows for greater spatial coverage than possible with in-situ methods.

A Simple or Abtew Method [16] is another technique that can provide lake evaporation estimates using solar radiation information. The method of potential ET, lake evaporation and wetland ET estimation has been successfully applied in South Florida [16-19]. Preliminary analysis shows that this model can be applied at other locations. The adjustments of the coefficient, *K* in the model for different regions can provide reasonable estimates of potential evapotranspiration.

Lake evaporation (E_o_) depends on the availability of energy and the mechanism of mass transfer, depth, and surface area of the lake. Evaporation is a function of solar radiation, temperature, wind speed, humidity, atmospheric pressure, and the surrounding environment. The most commonly used and the simplest method is the Pan method (Equation 1) where evaporation from large surface area lake is related to evaporation from a small pan. The common problems with the pan method are errors caused due to difference in environment between the pan and the lake and errors in pan evaporation measurement. The use of pan data requires the development of a coefficient (K_p_) to relate pan evaporation (E_pan_) to lake evaporation. As the settings and operations of pans differ, different pan coefficients would be required for each pan to relate it to a single lake's evaporation. Abtew, [19] developed pan coefficients for seven pans in South Florida correlating monthly pan evaporation to evaporation from Lake Okeechobee and developed annual average pan coefficients ranging from 0.64 to 0.95:

(1) $$E_{o}=K_{p}E_{\text{pan}}$$

Energy balance, mass and momentum transfer methods require measurement and estimation of several parameters and coefficients. The energy balance method requires input data of net radiation, sensible heat flux and change of energy storage in the lake. Mass transfer and momentum transfer methods need differences in specific humidity, wind speed at different heights and mass and momentum coefficients. Combination methods as the Penman and Penman-Monteith methods are input parameter intensive. The Penman-Monteith method input requirements include measured parameters solar radiation, air temperature, humidity, wind speed and atmospheric pressure. The method also requires derived parameters air density, canopy resistance, aerodynamic resistance, vapor pressure deficit, psychrometric constant, slope of saturation vapor pressure curve and heat storage. Additionally, estimated parameters as stomatal resistance, leaf area index, cover height, displacement height, aerodynamic roughness, momentum roughness height and heat capacity are needed for vegetated surfaces. Radiation and temperature based methods are less input data demanding.

### Lysimeter Study of Wetland Evapotranspiration and Open Water Evaporation

**Figure 1.**
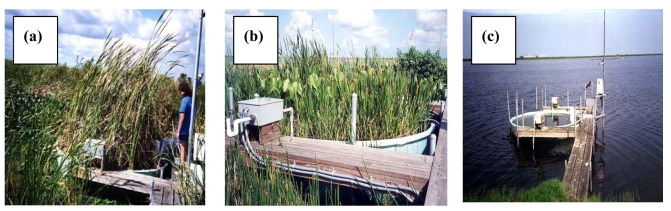
Automated lysimeters in three surface covers (a) cattails, (b) mixed marsh and (c) open water.

A two-year lysimeter study of evapotranspiration in three wetland environments (cattails, mixed vegetation marsh, and open water/algae) was conducted in the Everglades Nutrient Removal Project, a constructed wetland in south Florida (26° 38′ N, 80° 25′ W), USA. The study was conducted between 1993 and 1996 [16-20]. Figure 1 shows cattail, mixed vegetation, and open water lysimeters in the Everglades Nutrient Removal constructed wetland. The design of the lysimeter system is presented in Abtew and Hardee [21]. The results of the study were applied to test and calibrate six evapotranspiration estimation models: Penman-Monteith, Penman-Combination, Priestly-Taylor, Modified Turc, Radiation/Tmax, and Radiation (Simple) methods. It was indicated that the outputs from the Simple method was comparable to the observed potential evapotranspiration in the three different experimental studies. The Simple Method required a single measured parameter and achieved comparable performance to the complex methods with numerous input requirements.

## 2. Methodology

### 2.1. The Simple or Abtew Method

**Figure 2.**
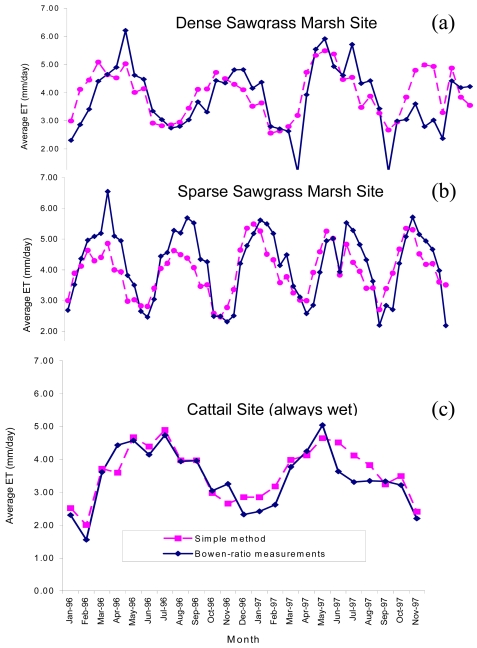
Comparison of the Simple (Abtew) Method ET with Bowen ratio measurements for south Florida wetland system: covers (a) dense sawgrass marsh, (b) sparse sawgrass marsh, and (c) cattail.

Input data requirements increase from the Simple Method to the Penman-Monteith Method. In South Florida, most of the variance (73 percent) in daily evapotranspiration is explained by solar radiation alone. The effect of humidity and wind speed in estimating ET is relatively minimal. The Simple Method (Equation 2) requires a single measured parameter, solar radiation, and is less subject to local variations [4]. The Simple Method is also cited as Abtew Equation and Simple Abtew Equation in published literature:

(2) $$\text{ET}=K_{1}\frac{\text{Rs}}{\lambda }$$

where ET is daily evapotranspiration from wetland or shallow open water (mm d^-1^), Rs is solar radiation (MJ m^-2^ d^-1^), λ is latent heat of vaporization (MJ kg^-1^), and K_1_ is a coefficient (0.53). The Simple Method was further cross validated by comparing the estimates to four years of Bowen-Ratio ET measurements at nine sites in the Everglades of South Florida [17]. Figures 2a, b and c show very good correspondence of Simple Method estimated and Bowen Ratio measured wetland ET.

**Figure 3.**
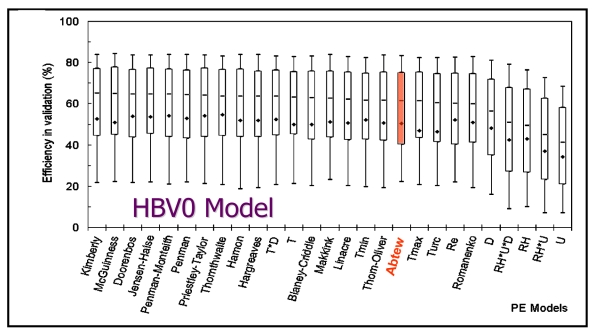
Average and 0.10, 0.25, 0.50, 0.75 and 0.90 percentile of Nash-Sutcliffee (E) Criteria obtained by 27 PE models in validation mode for HBVO model [22].

Comparative application of the Simple Method further demonstrates its usefulness. In an effort to identify the most relevant approach to calculate potential evapotranspiration (E_o_) for use in daily rainfall-runoff models, Oudin *et al.* [22] compared 27 potential ET models for stream flow simulation from 308 catchments in France, United States and Australia. Each potential ET model estimate was applied to four continuous daily lumped rainfall-runoff models. Comparison of the Nash-Sutcliffe [23] efficiency (Equation 3) in validation of various potential ET methods as applied in the HBVO model is shown in Figure 3. The Abtew Method has comparative efficiency to most models. The model efficiency (*E*) goodness-of-fit measure is based on the error variance and observed variance is, defined as:

(3) $$E=\left[1-\frac{\sigma_{ɛ}^{2}}{\sigma_{o}^{2}}\right]$$

The error variance, *σ^2^_ε_*, is defined as:

(4) $$\sigma_{ɛ}^{2}=\frac{1}{n-1}\sum_{i=1}^{n}{\left({\text{PE}}_{o,i}-{\text{PE}}_{p,i}\right)}^{2}$$

The variance of the observed potential evapotranspiration (PE), *σ^2^_o_*, defined as:

(5) $$\sigma_{o}^{2}=\frac{1}{n-1}\sum_{i=1}^{n}{\left({\text{PE}}_{o,i}-{\bar{\text{PE}}}_{o}\right)}^{2}$$

where, *PE_o_, PE_p_, PE_0_¯* are observed, predicted and average measured PE, respectively.

The Simple Method referenced as the radiative Abtew model, was applied to estimate evaporation from Lake Titicaca, South America. Compared to eight evaporation models, it was found to be the best evaporation estimation model [24]. Xu and Singh [25] evaluated various radiation based methods for calculating evaporation and concluded that the Simple Method referenced as the simple Abtew equation, can be used when available data is limited to radiation data. The Simple Method is applicable to remote sensing where the input, solar radiation, is acquired through satellite observations [26].

### 2.2. Remote Sensing Application in ET Estimation

Remote sensing-based ET estimations using the surface energy budget equation are proven to be one of the most recently accepted techniques for areal ET estimation covering larger areas [9]. Surface Energy Balance Algorithms for Land (SEBAL) is one of such models utilizing Landsat images and images from others sensors with a thermal infrared band to solve equation (6) and hence generate areal maps of ET [6-9].

SEBAL requires weather data such as solar radiation, wind speed, precipitation, air temperature, and relative humidity in addition to satellite imagery with visible, near infrared and thermal bands. SEBAL uses the model routine of ERDAS Imagine, an image processing software, in order to solve the different components of the energy budget equations. In the absence of horizontally advective energy, the surface energy budget of land surface satisfying the law of conservation of energy can be expressed as:

(6) $$R_{n}-\text{LE}-H-G=0\;({\text{W}/\text{m}}^{2})$$

where *R_n_* is net radiation at the surface, *LE* is latent heat or moisture flux (ET in energy units), *H* is sensible heat flux to the air, and G is soil heat flux. Energy flux models solve equation (6) by estimating the different components separately. Net radiation is estimated based on the relationship by Bastiaanssen [5]:

(7) $$R_{n}=R_{s↓}(1-\alpha )+R_{L↓}-R_{L↑}-R_{L↓}(1-{ɛ}_{s})\;({\text{W}/\text{m}}^{2})$$

where *R_S↓_* (W/m^2^) is the incoming direct and diffuse shortwave solar radiation that reaches the surface; *α* is the surface albedo, the dimensionless ratio of reflected radiation to the incident shortwave radiation; *R_L↓_* is the incoming long-wave thermal radiation flux from the atmosphere (W/m^2^); *R_L↑_* is the outgoing long-wave thermal radiation flux emitted from the surface to the atmosphere (W/m^2^), *ε_s_* is the surface emissivity, the (dimensionless) ratio of the radiant emittance from a greybody to the emittance of a blackbody.

The soil heat flux is the rate of heat storage to the ground from conduction. Studying irrigated agricultural regions in Turkey, Bastiaanssen [5] suggested an empirical relationship for *G* given as:

(8) $$G/R_{n}=0.2(1-0.98{\text{NDVI}}^{4})\;({\text{W}/\text{m}}^{2})$$

where *NDVI* is the normalized difference vegetation index (dimensionless).

Sensible heat flux is the rate of heat loss to the air by convection and conduction due to a temperature difference. Using the equation for heat transport, sensible heat flux can be calculated as:

(9) $$H=\frac{\rho C_{p}\left(T_{s}-T_{a}\right)}{r_{ah}}\;({\text{W}/\text{m}}^{2})$$

where *ρ* is the density of air (kg/m^3^), *C_p_* is the specific heat of air (1004 J/kg/K), *T_a_* is the air temperature (K), *T_s_* is surface temperature (K) derived from the thermal band of Landsat images and *r_ah_* is the aerodynamic resistance (s/m).

With *R_n_, G*, and *H* known, the *latent heat flux* is the remaining component of the surface energy balance to be calculated by SEBAL. Rearranging equation (6) gives the latent heat flux where:

(10) $$LE=R_{n}-G-H\;({\text{W}/\text{m}}^{2})$$

The detailed technique for estimating latent and sensible heat fluxes using remotely-sensed data from Landsat and other sensors is documented and was tested in Europe, Asia, Africa, and in Idaho in the US and proved to provide good results [5-8].

In this study, two Landsat images (Landsat TM from December 1986 and Landsat ETM+ from January, 2000) were used for the computation of energy fluxes and lake evaporation estimation. Images were processed based on the procedures outlined in SEBAL model. Based on the daily lake evaporation estimates from the SEBAL model, monthly estimates were generated for comparison to the Simple and Penman Models.

### 2.3. Surface Energy Balance Application at Glacial Ridge Wetland, Minnesota

**Figure 4.**
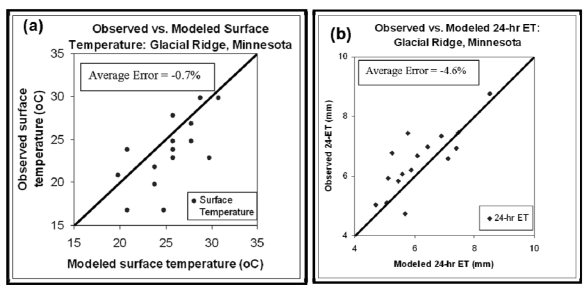
Observed *vs*. modeled (a) ET and (b) surface temperature from Glacial Ridge wetland, Minnesota using remote sensing.

The application of the Landsat imagery-based surface energy balance approach to wetland ET estimation was evaluated using 2000–2003 summer-time images for the Glacial Ridge wetland restoration area in Minnesota. Results of the analysis indicate that, the SEBAL-based 24-hr ET values correspond well with observed values with an average percent error of −4.6% (Table 1 and Figure 4a). The study also found that the Landsat-based surface temperature was comparable with observed values with an average error of −0.7% (Figure 4b). The result in the above study confirmed that, remote sensing-based approaches of ET estimation to be effective for wetlands.

## 3. Results and Discussion

### 3.1. Simple Method Application to Lake Ziway

**Figure 5.**
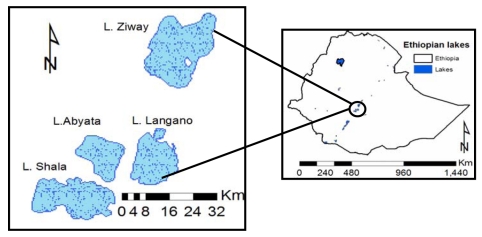
Ethiopian Rift valley lakes.

Lake Ziway (Figure 5) is located in the Ethiopian Rift valley with an average surface area of 490 km^2^ at an elevation of 1,636 m msl [27]. Monthly and annual average Lake Ziway evaporation estimates have been published [27,28]. The estimates vary from method to method of evaporation estimation. Annual lake evaporation estimates by Coulomb *et al.* [27] were 1,777, 1,875 and 1,728 mm, respectively estimated with the Energy balance, Penman and Complementary Relationship Lake Evaporation (CRLE) methods. Estimates by Ayenew [28] were 2,022, 1,599 and 1,769 mm, respectively, estimated with the Penman, Radiation and Pan Methods (Table 2).

**Figure 6.**
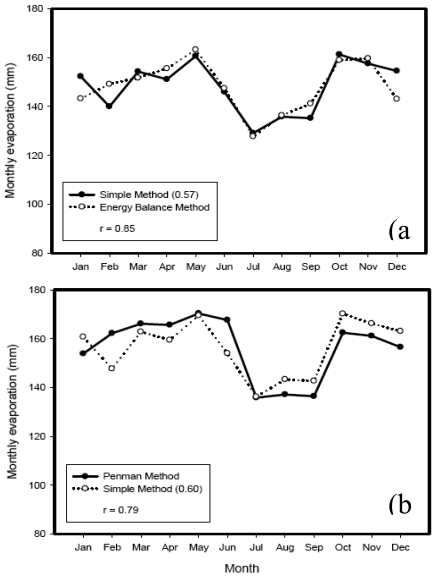
Comparison of models for estimates of Lake Ziway evaporation (a) Energy balance *vs*. Simple Method (b) Penman *vs*. Simple Method.

Application of the Simple Method was tested with input of average monthly solar radiation data [27]. With the original coefficient value (K = 0.53), the annual lake evaporation estimate by the Simple Method is 1662 mm (Table 2). The Simple Method estimate is 4 percent lower than the CRLE method estimates [27] and 3.8 percent higher than the Radiation method estimates [28]. The coefficient of the Simple Method can be adjusted to match annual estimates of any of the methods as a way of calibration if it is believed those methods are more reliable. Monthly lake evaporation estimates by the Energy Balance and Penman Equation [27] were compared to the Simple Method with adjusted K values. Results are shown in Figures 6a and 6b. Monthly lake evaporation estimates by the Simple Method with single measured parameter, solar radiation, has fitted well compared to estimates of the energy balance and Penman equation.

### 3.2. Surface Energy Balance and Remote Sensing Application to Lake Ziway

**Figure 7.**
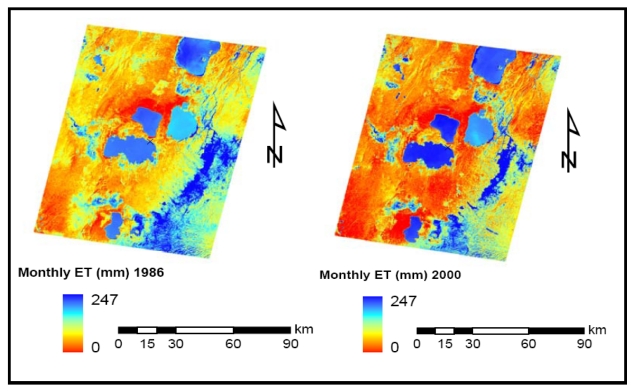
Spatial monthly evapotranspiration map of Rift valley lakes, Ethiopia.

Table 3 shows statistics of the results of the remote sensing-based lake evaporation estimates. It is shown that the monthly estimates correspond well with the long-term averages of the monthly evaporation values from the Simple and Penman Method for Lake Ziway. Comparison of lake evaporation estimates among lakes shows that, Lake Langano, the mercky lake with high sediment loads has lower average monthly evaporation than the other three lakes, which have less sediment loads, deeper and clearer than Lake Langano. The high surface thermal radiance of the turbid lake shows a higher surface temperature than the other clearer lakes which have less near surface absorption of solar radiation. The higher near surface temperature can be related to a lower ET than the other surfaces which are shown to be cooler. This can be one of the weaknesses of the surface energy balance approach using the thermal data from remote sensing. Figure 7 shows the spatial evapotranspiration estimates from the two Landsat images.

## 4. Summary

Evaporation from ponds, lakes and reservoir is a key hydrologic parameter and cost effective estimation method is desired specially in areas where monitoring resources are limited. Open water evaporation can be estimate of potential evapotranspiration. The Simple Method has been tested and applied in South Florida where it is currently the standard method for lake evaporation, wetlands evapotranspiration and potential evapotranspiration estimation by South Florida Water Management District, a 47,000 square km complex water management system. Applications of the method to other regions are also cited. The method should provide good evaporation estimate in tropical and subtropical areas where humidity is high, wind speed is not high and temperature is correlated to radiation. In this study the Simple Method evaporation estimates with and without recalibration of the coefficient, K, are comparable to the estimate of the input data intensive methods.

The application of the Simple Method and surface energy balance approach using remotely-sensed data were applied to Rift Valley Lakes of Ethiopia, where other approaches are less effective due to limited observed meteorological data and remoteness of the areas. The monthly lake evaporation estimates from the two approaches corresponds very well with Penman and energy balance approaches from previous studies.

## Figures and Tables

**Table 1.** Comparison of the surface energy balance approach and observed 24-hr reference ET at the Glacial Ridge wetland, Minnesota.

**Date**	**Path/Row**	**Sensor**	**ET_r24_ Reference** **(mm)**	**ET_24_ SEBAL** **(mm)**	**ET_24_ Error** **(%)**	*T_s_ Observed **(°C)**	**T_s_ SEBAL** **(°C)**	**T_s_ Error** **(%)**
Jun 5-00	30/27	ETM+	7.35	6.9	-6.1	19.8	23.8	-1.37
Jul 23-00	30/27	ETM+	6.58	7.13	8.3	24.8	27.8	-1.01
Aug 24-00	30/27	ETM+	5.1	5.08	-0.4	22.8	25.8	-1.01
Sep 10-00	29/27	TM	6.92	7.39	6.8	20.8	19.8	0.34
Jun 9-01	29/27	TM	8.76	8.52	-2.7	24.8	25.8	-0.34
Jul 10-01	30/27	ETM+	6.67	6.10	-8.5	22.8	29.8	-2.36
Aug 4-01	29/27	ETM+	5.93	5.12	-13.7	29.8	28.8	0.33
Aug 27-01	30/27	ETM+	5.84	5.47	-6.3	21.8	23.8	-0.68
Jun 4-02	29/27	ETM+	5.03	4.7	-6.6	16.8	24.8	-2.76
Jun 27-02	30/27	ETM+	6.19	5.9	-4.7	26.8	27.8	-0.33
Jul 29-02	30/27	ETM+	6.07	5.59	-7.9	23.8	25.8	-0.67
Sep 7-02	30/27	TM	6.97	6.43	-7.7	27.8	25.8	0.66
Jun 15-03	29/27	TM	7.45	7.46	0.1	23.8	20.8	1.01
Jul 24-03	30/27	TM	7.44	5.78	-22.3	23.8	25.8	-0.67
Aug 18-03	29/27	TM	6.76	5.26	-22.2	29.8	30.8	-0.33
Sep 3-03	29/27	TM	4.73	5.69	20.3	16.8	20.8	-1.38

**Average**			**6.5**	**6.2**	**-4.6**	**23.6**	**25.5**	**-0.7**

**Table 2.** Lake Ziway evaporation estimations with various methods.

**Month**	**Solar Radiation**	**Simple Method Lake Evaporation K = 0.53**	[27]			[28]		

			**Energy**	**Penman**	**CRLE**	**Penman**	**Radiation**	**Pan**

	W/M^2^	mm	mm	mm	mm	mm	mm	mm

January	246	142	143	154	132	150	135	159
February	250	131	149	162	135	128	120	144
March	249	144	152	166	149	148	142	192
April	252	141	156	166	155	188	138	142
May	259	150	163	170	160	188	138	156
June	243	136	147	168	155	135	107	137
July	208	121	128	136	146	139	115	126
August	219	127	136	137	136	135	115	123
September	226	126	141	136	137	164	124	112
October	260	151	159	162	141	200	151	170
November	263	147	160	161	144	223	157	178
December	249	145	143	157	139	225	157	130
**Total**		**1662**	**1777**	**1875**	**1728**	**2023**	**1599**	**1769**

**Table 3.** Monthly lake evaporation statistics (mm).

**Ziway**	**1986**	**2000**	**Langano**	**1986**	**2000**
**Min**	121.0	121.8	**Min**	110.0	100.4
**Max**	204.0	201.0	**Max**	194.0	199.0
**Mean**	145.0	138.7	**Mean**	116.9	115.0
**STD**	7.3	2.13	**STD**	1.6	4.1

**Abiyata**	**1986**	**2000**	**Shala**	**1986**	**2000**

**Min**	129.6	129.7	**Min**	131.2	130.5
**Max**	148.2	185.0	**Max**	192.0	196.0
**Mean**	140.9	141.8	**Mean**	135.5	143.7
**STD**	2.1	2.1	**STD**	1.3	7.8
